# Getting a grip on StAR-related lipid-transfer (START) domain proteins in bacteria

**DOI:** 10.1093/femsre/fuag042

**Published:** 2026-08-22

**Authors:** Ece Aslan, Ksenia I Lubova, Alex Speer, Wilbert Bitter

**Affiliations:** Medical Microbiology and Infection Control, Amsterdam UMC, Amsterdam, 1081 HZ, the Netherlands; Biomolecular Sciences and Biotechnology, University of Groningen, Groningen, 9747 AG, the Netherlands; Medical Microbiology and Infection Control, Amsterdam UMC, Amsterdam, 1081 HZ, the Netherlands; Medical Microbiology and Infection Control, Amsterdam UMC, Amsterdam, 1081 HZ, the Netherlands; Molecular Microbiology A-LIFE Department, Vrije Universiteit Amsterdam, Amsterdam, 1081 HZ, the Netherlands

**Keywords:** start domain, lipid transport, cyclase, *Mycobacterium*, ubiquinone, SRPBCC

## Abstract

StAR-related lipid transfer (START) domain proteins comprise a conserved superfamily defined by a characteristic helix-grip fold that enables the binding of hydrophobic ligands. In mammals, START domain proteins have been extensively characterized as key mediators of nonvesicular lipid transport and lipid-dependent signalling pathways. In contrast, the prevalence, structural diversity, and functional roles of START domain proteins in bacteria remain underexplored and for those that are characterized, experimental findings are occasionally conflicting. While bacterial START domains preserve the core helix-grip fold for lipid binding, they are typically smaller and exhibit more limited conformational flexibility than their eukaryotic counterparts. Despite these apparent constraints, available experimental data suggest that bacterial START domains participate in a remarkably broad range of biological processes—including small-molecule binding, metabolic regulation, enzymatic catalysis, and stress adaptation—rather than traditional lipid transport. Bacteria within the phylum Actinomycetota, in particular, have evolved a prolific repertoire of START-domain proteins. As an example, we will discuss the START domain proteins of *Mycobacterium tuberculosis* in detail, one of which has emerged as a promising drug target. Collectively, this synthesis underscores the functional versatility of the START domain across the domains of life and identifies critical knowledge gaps that warrant further investigation.

## Teaser

Bacterial START domain proteins have a conserved helix-grip fold, but diverge from mammalian lipid transporters by utilizing a more versatile array of processes, including metabolic regulation and secondary metabolite production.

## Introduction

StAR-related lipid transfer (START) domain proteins are named after the human steroidogenic acute regulatory protein-related lipid transfer, which is now known as STARD1 (Ponting and Aravind 1999). STARD1 facilitates the transport of cholesterol between the outer and inner membranes of mitochondria (Strauss et al. 2003, Lim and Cheon 2025). In the inner membrane of endocrine cells, cholesterol is then metabolized into a precursor for steroid hormone biosynthesis (Tugaeva and Sluchanko 2019, Lim and Cheon 2025). Because this transport step is rate-limiting in the production of steroid hormones, its role was deemed regulatory and hence the name *steroidogenic acute regulatory protein* (Clark et al. 1994). Soon after this, it was shown that other mammalian proteins similar to STARD1 are also involved in lipid transfer and a new protein family domain was born (Lavigne et al. 2010b, Clark Barbara and Stocco 2014). Another early discovered protein with a similar fold was a plant protein, Bet v 1 (Radauer et al. 2008). This protein was identified because it is plaguing a considerable part of humanity, as it is the primary allergen found in birch pollen (Faber et al. 1996, Breiteneder and Kraft 2023). These two founding members already indicate that START domain proteins can have very different roles, a feature that will be coming back in this review. In some literature the superfamily is called SRPBCC (after START/RHO_alpha_C/PITP/Bet_v1/CoxG/CalC), but we will use START domain as a general term for this family, as this is also used by the InterPro classification of proteins.

The START fold is now recognized as a versatile protein fold present in plants, yeast, mammals, and bacteria (Clark Barbara and Stocco 2014). START proteins bind a broad spectrum of hydrophobic ligands, including membrane lipids such as cholesterol, phosphatidylcholine (PC), phosphoinositide, ceramides, and fatty acids, but also hydrophobic compounds such as sterols, flavonoids, hormones, and ubiquinones (Ponting and Aravind 1999, Clark Barbara 2020). In principle, it can be stated that START-domain proteins provide a sheltered environment for hydrophobic compounds to move through aqueous environments (Fidler et al. 2022). These bound lipids are not only essential components of cellular membranes, but they also serve as signalling molecules, energy reservoirs, hormones, and enzyme cofactors and modulators of protein function (Chiapparino et al. 2016). Despite low sequence identity across the family, their helix-grip fold (see below) is structurally conserved, allowing the pocket to selectively capture hydrophobic molecules (Hurley and Tsujishita 2000, Iyer et al. 2001). START domains exist either as stand-alone soluble proteins or as modules within larger multidomain architectures (Soccio and Breslow 2003, Schrick et al. 2004, Mahtha et al. 2023). Because mammalian START family proteins are the best studied, they were initially viewed as the paradigm for the family’s molecular function. Despite their well-characterized roles in eukaryotes, bacterial START domain proteins remain poorly understood. Still, genomic studies show a large number of START domain proteins in bacteria, including both pathogenic and nonpathogenic species (Iyer et al. 2001, Schrick et al. 2004). In many organisms, these domains appear as isolated occurrences, yet their conservation across diverse phyla suggests that the START fold provides fundamental advantages in lipid binding, sensing or transfer. In *Streptomycetes*, they function as polyketide cyclase to produce secondary metabolites such as antibiotics (Thompson et al. 2004, Lee et al. 2012). We will also discuss the START domain proteins of *Mycobacterium tuberculosis*, where their presence is also greatly expanded. The broad evolutionary distribution underscores the need to integrate insights from both well-defined mammalian systems and emerging bacterial models. In this context, this review aims to summarize the structural and functional diversity of eukaryotic START domain proteins, with emphasis on the mammalian ones, and explore the exciting emerging evidence regarding bacterial START proteins.

## Mammalian START-domain proteins

In eukaryotic cells, lipid transport occurs through two primary routes: vesicular and nonvesicular. Vesicular transport involves the encapsulation of lipids within membrane-bound vesicles that bud from donor compartments and fuse with target membranes, allowing bulk delivery of lipids and proteins (Funato et al. 2020) .While this mode effectively supports large-scale lipid distribution and secretory trafficking, it lacks the precision and speediness required for certain contexts, particularly where local lipid composition must be tightly regulated in a short time span. Nonvesicular lipid transport operates via direct lipid exchange between membranes mediated by lipid-transfer proteins (Egea 2021). Increasing evidence shows that nonvesicular lipid transport is especially prominent at membrane contact sites (MCSs), regions where organelles such as the endoplasmic reticulum (ER), mitochondria, Golgi apparatus, peroxisomes, and endosomes are brought into close proximity without undergoing fusion (Lahiri et al. 2015, Cockcroft and Raghu 2018). Within these nanometre-spaced junctions, the lipid transfer proteins facilitate lipid transfer, coordinate lipid-dependent signalling pathways, and ensure efficient communication between organelles during metabolic shifts and stress responses. Among the nonvesicular lipid transport proteins, the START domain proteins represent a highly conserved, functionally adaptable superfamily that plays a central role (Alpy and Tomasetto 2005). Mammals have 15 START domain proteins, categorized into six subfamilies based on sequence phylogeny and ligand type (Clark Barbara 2020). Multidomain START proteins frequently couple the lipid binding to additional regulatory or membrane-targeting modules, such as PH domains, kinases, phosphatases, or FFAT (two phenylalanine residues in an acidic tract) motifs, thereby enabling lipid binding to allosterically catalytic regulation or scaffold lipid-dependent signalling pathways (Alpy and Tomasetto 2014). These architectures expand the functional repertoire of the START fold, allowing lipid binding to regulate enzymatic activity, scaffold signalling pathways or position the START domain precisely at organelle contact sites.

### Structure of START domain proteins

**Figure 1:**
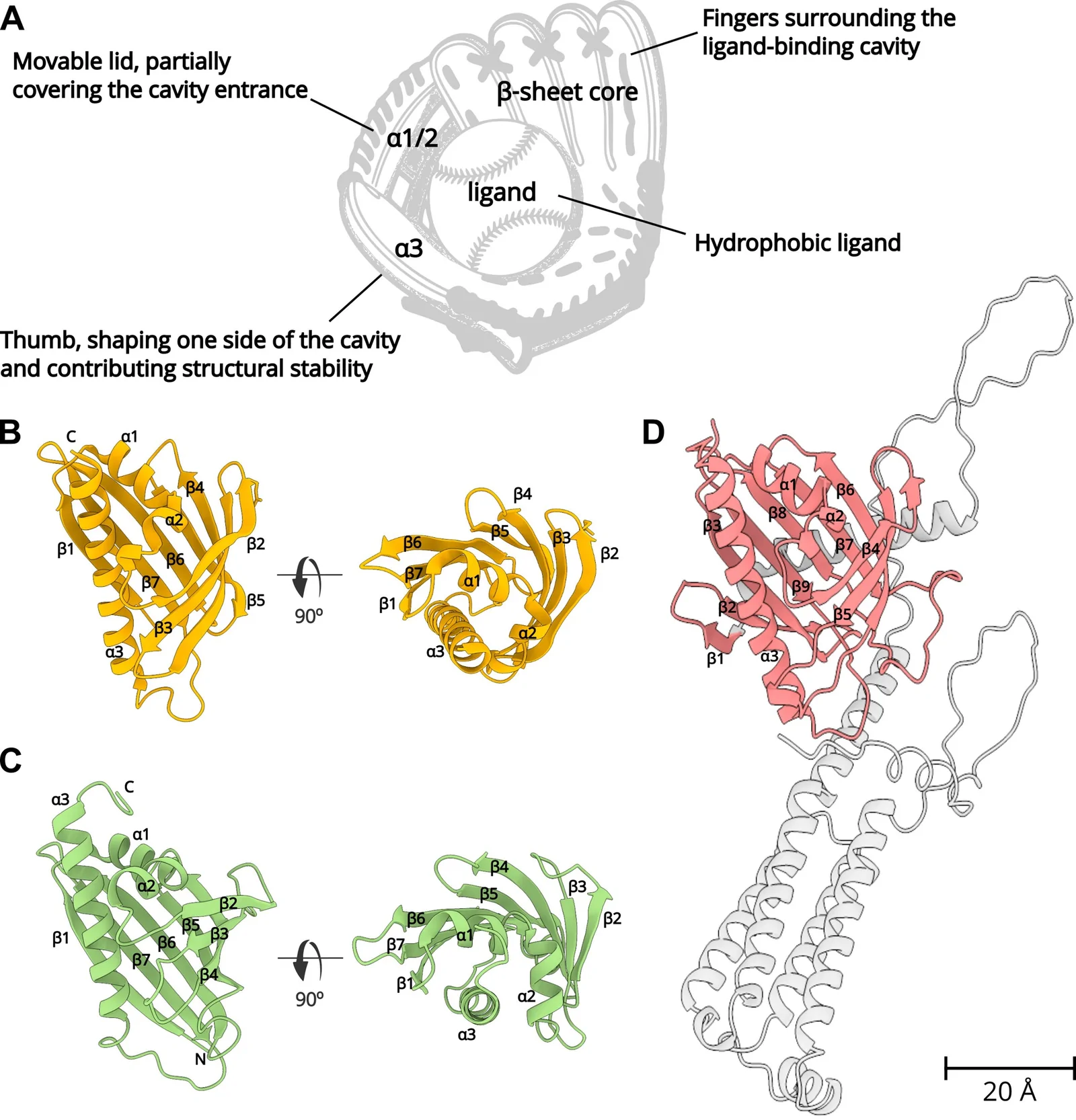
Structural overview and diversity of START-domain proteins. (A) Schematic representation of the START-domain fold using the analogy of a baseball glove. The curved antiparallel β-sheet forms the fingers, creating the rigid scaffold surrounding the ligand-binding cavity. The C-terminal α3 helix represents the thumb, shaping the cavity and contributing to ligand recognition and structural stability, while the N-terminal α1 and α2 helices function as a movable lid that partially covers the cavity entrance and regulates ligand access. (B–D) Representative domain organization and structures of bacterial, plant, and mammalian START-domain proteins. (B) Crystal structure of the hypothetical START-domain protein TTHA0849 from *Thermus thermophilus* (PDB: 2D4R). (C) Crystal structure of the birch pollen allergen Bet v 1 L (PDB: 1FM4). (D) Predicted structure of human STARD3 (AlphaFold model AF-Q14849-F1-model_v6). While bacterial START proteins typically consist of a single START domain, eukaryotic proteins frequently possess additional regulatory or membrane-targeting domains; the START domain is highlighted in light coral. Scale bar, 20 Å.

In mammals most START domains are ~200–210 amino acids (Ponting and Aravind 1999). The most distinctive feature of the START domain is its α/β helix-grip fold, which forms the core of its lipid-binding pocket (Hurley and Tsujishita 2000, Murcia et al. 2006, Clark Barbara 2020). This fold consists of a curved nine-stranded antiparallel β-sheet flanked by two main α-helices at the N- and C-termini (α1 and α4) and two short internal helices (α2 and α3), forming a compact globular structure. The C-terminal α-helix (α4) is most important and closes the groove that is formed by the β-sheet (Roostaee et al. 2009). Possibly, it functions as a dynamic lid that connects the β sheets and could regulate ligand access (Létourneau et al. 2014a, Husbands et al. 2023). This part of the structure resembles a baseball glove with 9 β-sheet fingers and the C-terminal α-helix as the thumb (Fig. 1A).

The concave face of the β-sheet and the C-terminal α4 helix together form a deep hydrophobic cavity, which serves as the principal lipid-binding site (Hurley and Tsujishita 2000, Létourneau et al. 2014b). The antiparallel β-sheet (β1–β9) wraps around the central cavity to form a cup-like structure, giving rise to the characteristic ‘helix-grip’ fold, where the C-terminal helix encircles and stabilizes the lipid ligand. The internal architecture of the hydrophobic pocket typically contains conserved aromatic and aliphatic residues, such as phenylalanine and leucine, that line the hydrophobic pocket, providing the van der Waals and hydrophobic interactions essential for the stabilization of the lipid ligand (Hurley and Tsujishita 2000, Romanowski et al. 2002.). Some START domains have been identified to have polar residues positioned near the entrance or the base of the pocket as well, allowing hydrogen bonding or electrostatic interactions with lipid headgroups (Hurley and Tsujishita 2000). In addition to this central structural motif, there are two short α-helices (α2 and α3) between β-sheets 3 and 4 that contribute to the structural integrity of the hydrophobic cavity and close it off at one end. Finally, the N-terminal α-helix (α1) stabilizes the β-sheet from the other side and does not contribute to the hydrophobic cavity.

**Figure 2:**
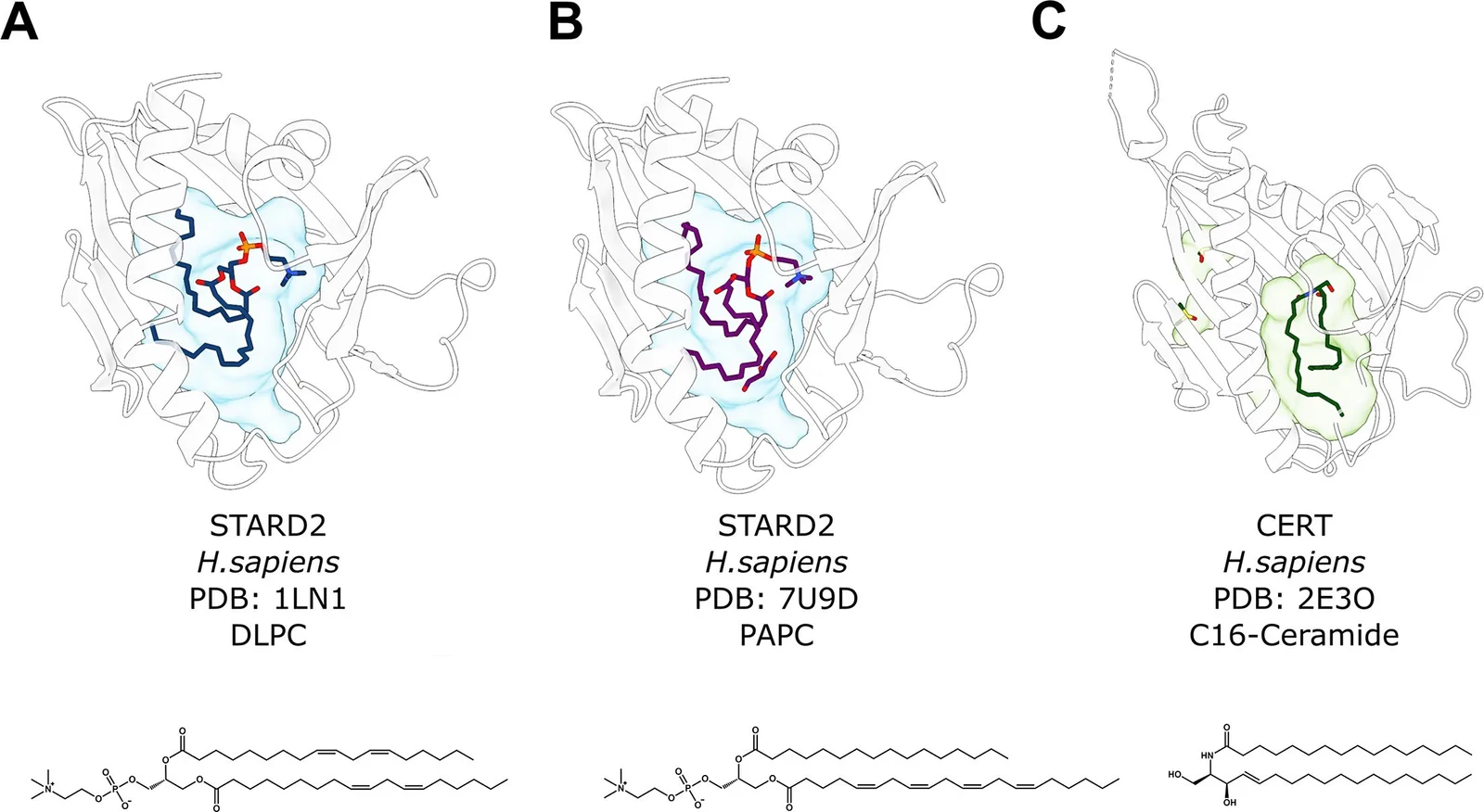
Ligand-bound structures of mammalian START-domain proteins. Experimentally determined structures are shown in grey, with ligand-binding cavities displayed as molecular surfaces and the corresponding ligand structures shown below each protein. (A) Human STARD2 bound to 1,2-dilinoleoyl-Sn-glycero-3-phosphocholine (DLPC; PDB: 1LN1). (B) Human STARD2 bound to 1-palmitoyl-2-arachidoyl-sn-glycero-3-phosphocholine (PAPC; PDB: 7U9D). (C) Human CERT bound to C16-ceramide (PDB: 2E3O). Cavities were visualized using parKVFinder v1.2.0 (Guerra et al. 2020).

Structural crystallography studies have revealed multiple apo- and ligand-bound forms of the mammalian START domain proteins, a list of which can be found in Table S1. STARD2/PC-TP has been predominantly characterized in ligand-bound conformations (Fig. 2). Comparative analyses across some of these structures demonstrated that, despite the low sequence identity, the tertiary fold is highly conserved (Schrick et al. 2004, 2014, Thorsell et al. 2011). When the crystal structures of the four START domains (STARD1, STARD5, STARD13, and STARD14) are compared and superimposed, their three-dimensional alignment reveals a root mean square deviation of ~2.3 Å across 154 residues that form the structural backbone of the characteristic helix-grip fold (Thorsell et al. 2011).

**Figure 3:**
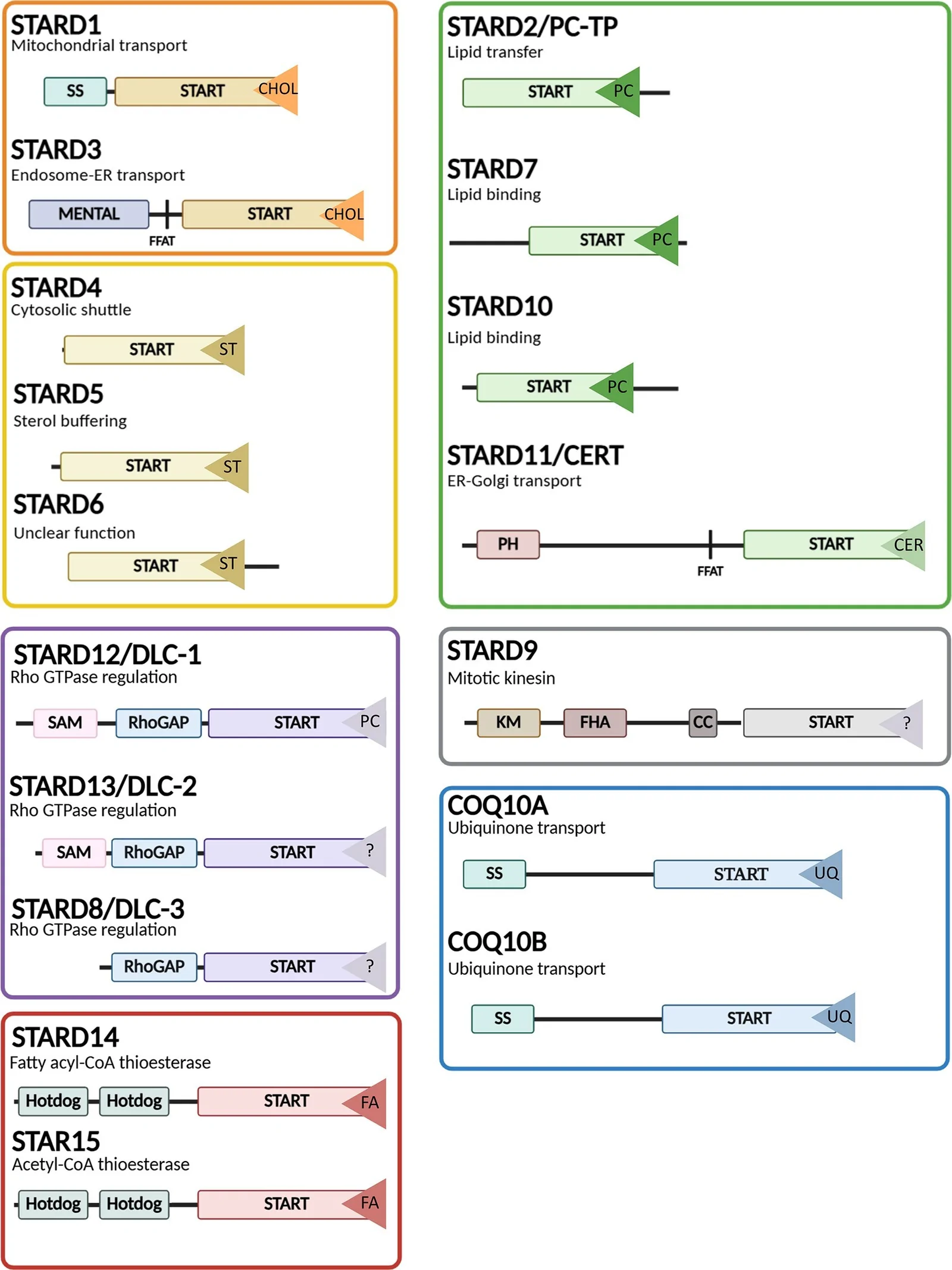
Summary of mammalian START proteins. Schematic representation of the domain organization of the human START domain protein family, grouped by structural/functional subfamily (coloured boxes). For each protein, a brief functional annotation is given beneath the gene name, and the corresponding domain schematic is shown below with the START domain depicted as an rectangle (shading by subfamily) whose principal ligand or ligand class is indicated within the rectangle: cholesterol (CHOL), sterol (ST), PC, ceramide (CER), fatty acid (FA), ubiquinone (UQ), or unknown (?). Accessory domains include SS (mitochondrial targeting sequence), MENTAL (membrane-anchoring domain), SAM (sterile alpha motif), RhoGAP (Rho GTPase-activating protein), Hotdog (thioesterase fold), PH (pleckstrin homology), KM (kinesin motor), FHA (forkhead-associated), and CC (coiled-coil). The FFAT motif (two-phenylalanines-in-an-acidic-tract) is marked where present.

The mammalian START domain proteins vary in their domain organization, which results in a collection of different cellular functions (Fig. 3). STARD1, for example, is extended at the N-terminus with a mitochondrial targeting sequence, which is cleaved off during transport. This directs STARD1 to the mitochondrial intermembrane space, where it facilitates cholesterol transport between the two membranes (Clark et al. 1994). By contrast; STARD3, also known as MLN64, is a multidomain protein in which the START domain is coupled to a MENTAL domain composed of four transmembrane helices that anchors the protein to late endosomal membranes followed by a FFAT motif (two phenylalanine residues in an acidic tract) that drives the ER contact site formation (Alpy et al. 2013, Wilhelm et al. 2017). The sterol-binding proteins STARD4, STARD5, and STARD6 exist as single soluble START domain folds, which allow them to dynamically associate with different membranes and function as transporters (Soccio and Breslow 2003). Within the phospholipid and ceramide-binding branch of the family, STARD2 similarly exists as a single START domain protein, whereas STARD11 (CERT) contains both an N-terminal PH domain and a FFAT motif that mediates anchoring to the ER (Kanno et al. 2007, Kumagai and Hanada 2019).

Finally, several mammalian START domain proteins are smaller and more closely resemble the originally defined helix-grip fold, typically comprising five to seven antiparallel β-strands flanked by two to three α-helices; an architecture shared with other helix-grip fold proteins such as the pollen allergen Bet v 1 (seven strands, three helices) (Gajhede et al. 1996) and, in its most minimal form, the TATA-binding protein (five strands, an N-terminal helix) (Ponting and Aravind 1999, Iyer et al. 2001), as well as most bacterial START domain proteins (as discussed in a later section). These proteins, COQ10a and COQ10b are often overlooked in the discussion of mammalian START domain proteins. They are chromosomally encoded but transported to the mitochondrion where they play a role in the binding and distribution of the ubiquinone coenzyme Q10 (Tsui HS et al. 2019). Coenzyme Q10 is an essential redox-active lipid functioning in the electron transport chain (Guerra and Pagliarini 2023). The corresponding genes of COQ10 are conserved in all major eukaryotic lineages and they possibly originate from the bacterial endosymbiont that evolved into mitochondria (Awad et al. 2018, Wang et al. 2024). A mutation or deletion of the COQ10 gene in yeast (*Saccharomyces cerevisiae*) results in a severe respiratory deficiency, characterized by an inability to grow on nonfermentable carbon sources due to a defective electron transport chain (Barros et al. 2005a). Yet, these yeast cells were shown to have normal amounts of mature ubiquinone (which is coenzyme Q6 rather than coenzyme Q10 in yeast). The model is that this START domain protein is required to function as a chaperone for the transport of Coenzyme Q from the site of synthesis to the electron transport chain (Barros et al. 2005b). Together, this diversity in the protein architecture across the START protein family shows how the conserved lipid-binding fold has been functionally adapted to operate within different environments and lipid trafficking pathways.

### Determinants and models of lipid binding

Although the START domain proteins show a relatively rigid β-sheet core, the C-terminal helix and the surrounding regions exhibit conformational flexibility that results in lipid binding and specificity (Thorsell et al. 2011, Alpy et al. 2013, Létourneau et al. 2016, Tugaeva and Sluchanko 2019). Tsujishita and Hurley (2000) solved the apo structure of MLN64/STARD3 and proposed that the C-terminal helix acts as a dynamic lid, with lipid entry occurring through transient opening of the Ω-loops and α4 helix. In the ‘open’ state, the C-terminal helix is displaced or partially unfolded, exposing the pocket and allowing lipid entry (Schrick et al. 2004, Clark Barbara 2020). Molecular dynamics simulations and crystallography studies of STARD1 and STARD3 have shown that this state is not fully static but rather dynamic, a principle likely applicable across the START family (Hurley and Tsujishita 2000, Murcia et al. 2006).

Local fluctuations of the Ω1-loop, C-terminal helix and the surrounding loops widen the cavity, which eventually creates a gating mechanism for lipid access (Murcia et al. 2006, Kudo et al. 2010). Once a ligand enters the START cavity, the C-terminal α4 helix acts as a dynamic lid closing over the hydrophobic pocket to stabilize the bound molecule and shield it from the aqueous environment (Hurley and Tsujishita 2000, Schrick et al. 2004, Tugaeva and Sluchanko 2019). These coordinated conformational changes are thought to be essential for regulating ligand entry, binding, and release across the START family, allowing the domain to accommodate a range of lipid types with controlled specificity (Létourneau et al. 2014a, b). This closure is not rigid; small adjustments in the α-helix and surrounding loops allow the pocket to accommodate differences in ligand shape and size. These coordinated movements effectively turn the START domain into a molecular shuttle, capable of transporting hydrophobic ligands while protecting them from the polar cytosol. The large flexible Ω1 loop, located between β5 and β6, may also contribute to shielding the hydrophobic pocket and regulating access to the cavity, providing an alternative pathway for ligand entry or exit (Murcia et al. 2006, Tugaeva and Sluchanko 2019).

Over the years, several mechanistic models have been proposed to describe lipid entry into the START-domain binding pocket, including the cavity complementarity (lock and key), the induced-fit, the intermediate state, and the Ω1 loop and α3 helix gating models (Létourneau et al. 2014a). These models differ in the proposed flexibility of the different structures, i.e. α4 and Ω1 that regulate the opening of the hydrophobic cavity, but they are partially overlapping. In the cavity complementarity model, ligand binding is largely dictated by geometric and chemical matching between the lipid and a preformed hydrophobic pocket, with minimal structural rearrangement (Murcia et al. 2006, Talandashti et al. 2024). The observation that the shape of the STARD1 cavity perfectly matches the cholesterol molecule implies that only limited structural changes are expected upon ligand binding, supporting the idea that selectivity is based primarily on the volume and shape complementarities between the ligands and the binding sites (Létourneau et al. 2014a). The induced fit and gating models emphasize the role of conformational flexibility in the C-terminal α4 helix, Ω1 loop, and α3 helix, proposing that transient structural rearrangements facilitate ligand entry and exit (Murcia et al. 2006, Kudo et al. 2010, Tugaeva and Sluchanko 2019). Finally, the intermediate-state model suggests that partial unfolding of the distal region of the α4 helix generates a short-lived ligand-accessible conformation before refolding stabilizes the bound molecule (Roostaee et al. 2009). Rather than representing mutually exclusive mechanisms, these models likely describe overlapping aspects of a shared dynamic binding process, with different START family members relying to varying extents on cavity geometry, helix and loop flexibility, and transient intermediate states to achieve ligand recognition and specificity.

No single model fully explains lipid binding across all START family members. Current evidence suggests that START domains use a variety of mechanisms for lipid binding, ranging from preformed pockets to intermediate states. Yet, major gaps in research are still present: (i) what triggers the lid opening during lipid release, (ii) which membrane interactions influence these transitions, and (iii) whether intermediate states like those proposed for STARD1 exist for other START proteins. Additionally, it is important to note that the conformational changes central to ligand entry have not been captured at high resolution. A more unified mechanistic model of lipid binding within the START domain family is essential for clarifying how ligand release is triggered, how conformational transitions are coordinated, and how these processes are regulated in different cellular environments. The structural determinants of ligand specificity are better characterized: although START domains share a common α/β helix-grip fold, variations in hydrophobic pocket volume, surface charge, and entrance polarity underlie the selective recognition of specific lipid types (Alpy and Tomasetto 2014).

Analyses of the apo crystal structures (ligand-free) of human STARD1, STARD4, STARD5, STARD13, and STARD14 reveal that the binding pockets of these proteins have their volume optimized for their (hypothesized) natural ligands, with each START domain displaying a cavity volume that corresponds to the molecular dimensions of the lipid it is proposed to bind (Lavigne et al. 2010b, Thorsell et al. 2011). For instance, crystal analysis on STARD11/CERT domain demonstrated that the hydrophobic cavity acts as a chain-length ‘ruler’ due to its volume, fully able to recognize C14–C20 ceramides, while excluding longer species (Kudo et al. 2008).

Surface charge and entrance polarity further plays a role in the ligand specificity of START domain proteins. Variation in the electrostatic environment around the binding pocket entrance influence the initial ligand recognition by favouring interactions with the lipid headgroups of the same polarity and optimizes the alignment of the lipid ligand towards the binding pocket (Kudo et al. 2008, Kang et al. 2010). An example of this can be seen in cholesterol-binding START domains in which the hydroxyl group of the cholesterol is oriented toward the polar and charged residues at the pocket entrance, enabling favourable electrostatic and hydrogen bonding interactions that guide the sterol into the cavity (Rodriguez-Agudo et al. 2008a, Lavigne et al. 2010a). Together, electrostatic complementarity and membrane-assisted ligand orientation not only guide the ligand into the correct position but also enhance its affinity and ensure a cavity environment that is selectively matched to the natural lipid of each START domain (Alpy and Tomasetto 2005).

**Figure 4:**
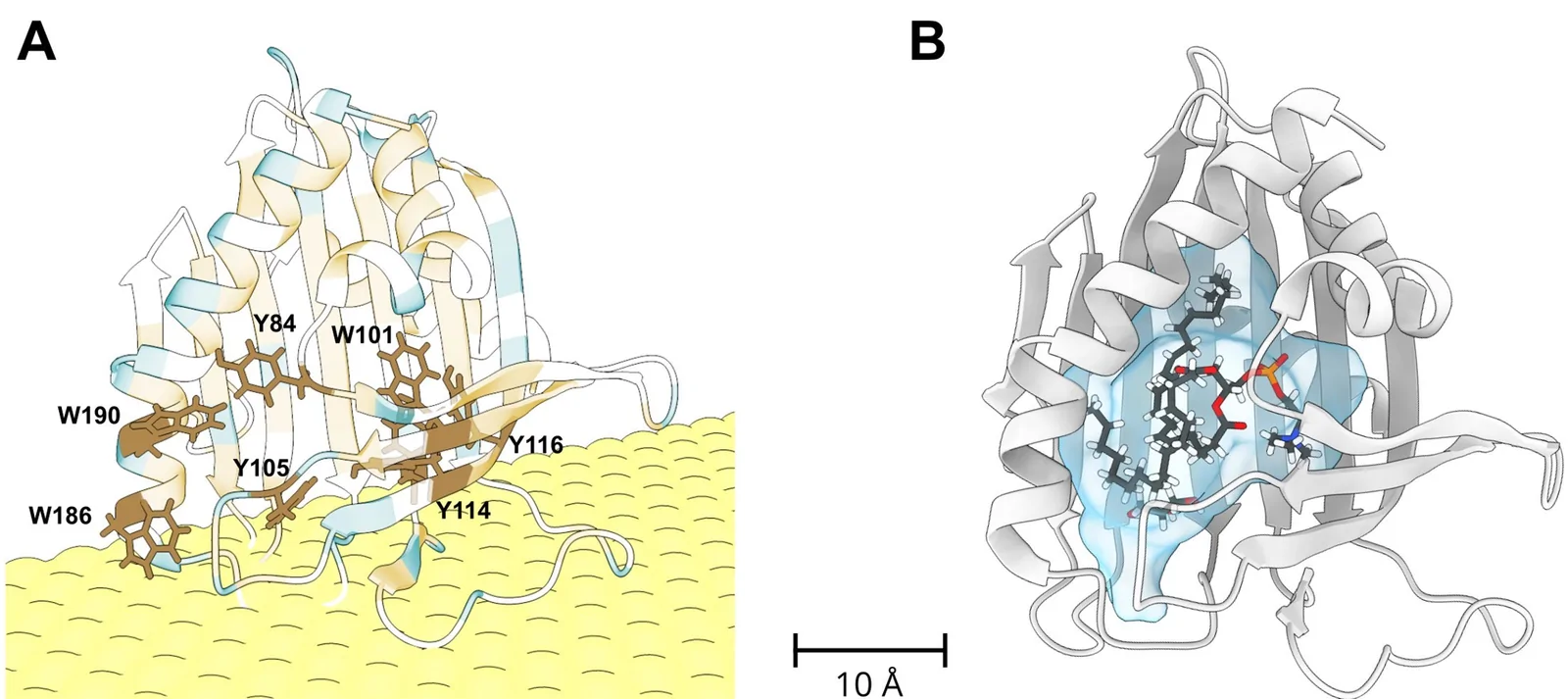
Proposed mechanism of phospholipid extraction by the mammalian START protein STARD2 (PC-TP). (A) Schematic representation of the membrane-binding and PC uptake mechanism of STARD2 based on molecular dynamics simulations. Upon transient association with the phospholipid bilayer, a series of conserved aromatic residues (Trp186, Trp190, Tyr84, Tyr105, Trp101, Tyr114, Tyr116, and Tyr155) mediate stepwise interactions with the phosphocholine headgroup through cation–π interactions, facilitating lipid extraction from the membrane and transfer into the hydrophobic binding pocket. (B) Structure of STARD2 in complex with PC (PDB: 7U9D). The ligand-binding cavity is shown as a blue surface, illustrating complete enclosure of the phospholipid within the START-domain pocket. Cavity was visualized using parKVFinder v1.2.0. Model from Talandashti et al. (2024).

STARD2 binds PC in a tunnel-like pocket. In the holo structure, the phosphate group is coordinated by Tyr72, Arg78, and Gln157, while the choline moiety is enclosed within an aromatic cage formed by Trp101, Tyr114, Tyr116, and Tyr155, fully sequestering the ligand within the START-domain pocket. Molecular dynamics simulations propose a stepwise uptake mechanism in which transient membrane association engages Trp186 and Trp190, followed by Tyr84 and Tyr105; these residues form successive choline–aromatic cation–π interaction sites that may facilitate PC extraction from the bilayer and guide the headgroup towards the final aromatic cage (Talandashti et al. 2024). These residues precisely position and stabilize PC, enabling selective recognition of its natural ligand by STARD2/PC-TP (Fig. 4). Crystal structures of the STARD11/CERT START domain bound to ceramides of different chain lengths show that the unsaturated sphingosine chain and saturated *N*-acyl fatty-acid chain are surrounded by hydrophobic residues lining the cavity, while the polar ceramide headgroup is stabilized by a hydrogen-bond network involving Arg442, Glu446, Gln467, Tyr482, Asn504, and Tyr553 (Kudo et al. 2008). The size and shape of the cavity contribute to chain-length selectivity, while the polar network helps position the ceramide headgroup within the pocket. The cavity is formed primarily by the α4 helix and Ω4 loop on one side, and the α3 helix and Ω1 loop on the other; the α3 helix and Ω1 loop have been proposed to function as a gate for ligand entry, although the available ligand-bound and apo crystal structures do not directly resolve an open-gate transition.

Most of the evidence on ligand specificity exists in docking and molecular dynamics results, yet these approaches come with limitations. Although simulations have been valuable for proposing mechanisms of ligand entry, loop rearrangements and pocket flexibility, they often do not fully replicate the complexity of ligand recognition observed in crystallographic studies (Romanowski et al. 2002, Alpy and Tomasetto 2014, Létourneau et al. 2014a, 2016). As a result, they require validation from experimentally derived structures. What all these models do show is that ligand specificity in START domains arises from a multitude of factors ranging from cavity volume, residues at the entrance of the binding pocket and flexibility of the structural elements.

### Mechanism of lipid transport by START domain proteins

Nonvesicular lipid transport by START domain proteins follow a conserved cycle in which the protein engages with a donor membrane, the hydrophobic cavity captures the lipid cargo, diffuses to an acceptor membrane and releases the cargo, with variations in the cargo specificity and release mechanisms (Hurley and Tsujishita 2000, Xie and Weinstein 2023, Moqadam et al. 2024). START-mediated lipid extraction begins when the protein engages with a donor membrane. This interaction is frequently guided by membrane-targeting domains, like the PH domains in STARD11/CERT or FFAT motifs in STARD3, or alternatively it has been suggested that it could also be accomplished through weak and intrinsic interactions between the START domain and the membrane (Alpy et al. 2013, Kumagai and Hanada 2019, Iaea et al. 2020). The details of cargo capture and release differ between sterol carriers and more complex lipid carriers, such as STARD11/CERT and STARD2/PC-TP, giving rise to two proposed mechanistic models for transport and release.

For sterol-transporting START proteins, the hydrophobic tunnel is preshaped to accommodate a single sterol, and the Ω1 loop gate opens to allow the ligand to enter, without requiring a specific membrane trigger (Hurley and Tsujishita 2000, Mesmin et al. 2013a). Molecular dynamics show that once bound, the gate closes and the protein acts as a direct shuttle, diffusing through the cytosol or the intermembrane space (in the case of STARD4) to deliver its cargo to the acceptor membrane (Xie and Weinstein 2023). The release step is thought to be driven by the thermodynamic favourability of inserting the hydrophobic sterol into the acceptor membrane’s interior, although this ignores the fact that this reaction will involve an intermediate state that is energetically less favourable. Furthermore, the direct shuttle model likely oversimplifies *in vivo* conditions (Alpy et al. 2013, Clark Barbara 2020). Membrane context, protein-protein interactions and local lipid composition can and will all modulate gate dynamics and transfer efficiency.

In contrast to the direct shuttle model, lipid carriers such as STARD11/CERT and STARD2/PC-TP are suggested to utilize a membrane-assisted allosteric release mechanism. With this mechanism, START domains first anchor themselves to the ER or Golgi membrane, stabilizing an open conformation that exposes the hydrophobic binding pocket to the lipid core of the membrane (Moqadam et al. 2024, Talandashti et al. 2024). This arrangement allows lipid tails, such as those of ceramide or PC, to partially insert into the pocket, effectively ‘greasing’ the cavity for the entry of the rest of the ligand. The combined effects of gate opening, anchoring and partial lipid tail insertion lowers the activation barriers for release, which results in the lipid cargo to sliding into the acceptor bilayer without requiring additional large-scale/major conformational changes from the START domain itself (Moqadam et al. 2024, Talandashti et al. 2024). The membrane-assisted allosteric release model provides a compelling mechanistic framework, but it relies heavily on microsecond time-scale simulations and limited experimental validation. Local lipid composition, enzymatic conversion and potential hand-off pathways may influence cargo release *in vivo*.

Cholesterol-binding mammalian START domain proteins (STARD1, STARD3, STARD4, STARD5, and STARD6) share the common function of transporting or sensing sterols, but each operates in a distinct biological context. STARD1 is the most extensively studied, known for its role in mediating cholesterol transfer within mitochondria to initiate steroid hormone synthesis (Elustondo et al. 2017). STARD4 and STARD5 function primarily in intracellular cholesterol trafficking to maintain lipid homeostasis in the plasma membrane and the ER (Rodriguez-Agudo et al. 2008b, 2019). STARD3, stands out for its involvement in endosome–ER contact sites and its role in cholesterol transport within cells, providing a link between membrane dynamics and lipid transport (Wilhelm et al. 2017). The physiological role of the final cholesterol-binding START protein, STARD6, is still unclear, despite its demonstrated capacity to bind and transport cholesterol metabolites (Létourneau et al. 2016).

Recent studies have revealed that the functional importance of STARD3 extends beyond simple sterol extraction. STARD3 is now recognized as a key mediator of endosome–ER contact sites, functioning through a FFAT motif that binds ER-resident VAP-A/B proteins and establishes stable, regulated MCSs (Wilhelm et al. 2017). These contacts bring late endosomes and the ER into nanometer-range proximity, enabling direct transfer of cholesterol from the endosomal membrane to the ER bilayer without requiring vesicular intermediates (Alpy et al. 2013, Wilhelm et al. 2017, Eichler et al. 2026). Within this confined space, the START domain can rapidly cycle between donor and acceptor membranes. The MENTAL domain further supports this process by concentrating sterols at the donor membrane surface, thereby increasing transfer efficiency (Wilhelm et al. 2017). Together, the combined anchoring, sensing, and tethering architecture of STARD3 enables a highly directed and spatially constrained form of lipid transfer.

In contrast to the membrane-anchored and MCS-driven mechanism of STARD3, STARD4 operates as a highly dynamic, soluble sterol shuttle (Talandashti et al. 2024). STARD4 lacks any transmembrane segments, FFAT motifs or other canonical targeting sequences, allowing it to diffuse freely throughout the cytosol. Its interactions with membranes are transient and mediated primarily by electrostatic contacts, particularly with negatively charged lipids such as phosphatidylserine (Mesmin et al. 2013b, Iaea et al. 2020). These weak, reversible interactions enable STARD4 to rapidly sample multiple organelles including the plasma membrane, endosomes, and the Golgi, extract a single cholesterol molecule into its hydrophobic cavity, and deliver it to the ER. This mobility gives STARD4 a different functional profile from STARD3: rather than directing sterol along a specific route, STARD4 utilizes sterol pools on a wider range. Loss-of-function studies demonstrated that without STARD4, sterol movement from the plasma membrane to the ER is significantly delayed, leading to inappropriate activation of cholesterol biosynthesis genes and disruption of lipid homeostasis (Rodriguez-Agudo et al. 2008b, Mesmin et al. 2013b, Iaea et al. 2020). The related protein STARD5 provides a buffering capacity, though with distinct ligand preferences. While STARD5 binds cholesterol, it also exhibits high affinity for oxysterols and bile acids, particularly under metabolic stress conditions, suggesting specialized roles in hepatic and immune contexts (Rodriguez-Agudo et al. 2005, 2019). Together, STARD4 and STARD5 form a flexible cytosolic system that integrates membrane sterol status with transcriptional and metabolic responses.

While cholesterol-binding START proteins orchestrate sterol homeostasis across endosomes and the ER and within mitochondria, a parallel set of START proteins operates within the equally essential network of glycerophospholipid and sphingolipid metabolism. Within this branch, STARD7 and STARD10 bind phospholipids or sphingolipids, STARD11/CERT binds ceramide and STARD2/PC-TP binds PC (Soccio and Breslow 2003, Alpy and Tomasetto 2005, 2014). STARD11/CERT is an example of how a START domain protein can integrate a multidomain architecture with nonvesicular lipid transfer. As a multidomain protein, CERT contains an N-terminal pleckstrin homology (PH) domain that selectively binds the phosphatidylinositol-4-phosphate-rich trans-Golgi membrane, a central FFAT motif enabling interaction with ER resident VAP proteins and a C-terminal START domain that selectively binds ceramide (Hanada et al. 2003, Kawano et al. 2006, Kumagai et al. 2007). Through these domains, CERT is positioned precisely at ER–Golgi contact sites, forming a molecular bridge that localizes the START domain to the spatial niche where ceramide that is produced in the ER must be delivered to the Golgi for its conversion into sphingomyelin. The conversion of ceramide into sphingomyelin is essential because it prevents the accumulation of ceramide, a potent mediator of stress and apoptotic signalling, while simultaneously generating a sphingolipid required for membrane stability and secretory pathway function (Chung et al. 2021, Taniguchi and Okazaki 2021). By directing ceramide to the Golgi for sphingomyelin synthesis, CERT ensures that cells maintain both lipid compositional balance and proper regulation of sphingolipid-dependent signalling pathways (Yamaji et al. 2008, Kumagai and Hanada 2019).

Mechanistically, CERT’s mode of action is best described as a membrane-guided lipid hand-off rather than a shuttle (Hanada et al. 2003). Biophysical and simulation studies show that ceramide release is strongly influenced by the local phospholipid environment of the acceptor membrane (Moqadam et al. 2024). In particular, PC in the trans-Golgi destabilizes the ceramide-bound state of CERT, effectively priming the complex for release. This membrane-assisted release creates a situation in which CERT does not simply “drop” ceramide into the membrane but rather coordinates its release in response to the lipid packing and composition of the acceptor bilayer (Moqadam et al. 2024). Once delivered, ceramide is rapidly converted to sphingomyelin, effectively pulling the reaction forward and maintaining a steep metabolic gradient that sustains directional transport.

Where CERT is a multidomain protein adapted for directional lipid flux at highly organized contact sites, STARD2 (PC-TP) represents the minimalist counterpart, a soluble, cytosolic phospholipid transfer protein that responds to membrane composition and metabolic cues with more mobility. STARD2 binds and transfers PC, but unlike CERT, it lacks membrane-targeting domains and instead relies on low-affinity interactions with organelle surfaces to capture and deliver lipid cargo (Roderick et al. 2002b, Kanno et al. 2007). PC transfer is vital because PC constitutes the dominant structural phospholipid of eukaryotic membranes; its redistribution governs membrane fluidity, organelle identity, and the availability of substrates for lipid remodelling. By equilibrating PC between organelles, STARD2 contributes to metabolic coupling and ensures that membrane composition can adapt to changes in growth, stress, or bioenergetic demand (Kanno et al. 2007, Kang et al. 2010, Talandashti et al. 2024). Recent mechanistic work has clarified how STARD2 functions in the absence of stable docking interactions. Simulations and biochemical assays suggest that STARD2 engages membranes through shallow, reversible contacts that allow the acyl chains of PC to partially insert into the protein’s lipid-binding cavity (Talandashti et al. 2024). This partial insertion appears to be the key activation step that triggers the conformational flexibility required for full lipid capture.

### Plant START domain proteins

As noted in the introduction, the birch pollen allergen Bet v 1 was one of the earliest identified START domain proteins. Although its exact physiological role remains unclear, Bet v 1 is a conserved plant stress-response protein belonging to the PR-10 family (Breiteneder and Kraft 2023b) and is known to bind the flavonoid compound quercetin-3-O-sophoroside (Seutter von Loetzen et al. 2014). Beyond this allergen, plant START domain proteins fall primarily into two main classes. The first class contains the HD-Zip Transcription Factors, DNA-binding homeodomain proteins containing a leucine zipper motif (Zheng et al. 2026). As homeobox transcription factors, these proteins act as master regulators of plant development. Two specific subfamilies, HD-Zip III and HD-Zip IV, feature a START domain and respond to phospholipids and sterols, respectively (Zheng et al. 2026). These multidomain proteins integrate several distinct functional regions.

The second class is formed by the abscisic acid receptors, which are probably of greater interest for this review as they are single-domain START proteins (Joshi-Saha et al. 2011). Abscisic acid functions as a vital stress hormone in plants, orchestrating essential survival responses, such as rapid stomatal closure to conserve water under severe drought, high salinity, and temperature extremes. Abscisic acid is bound by intracellular START domain receptors of the PYR/PYL/RCAR family. Although these START domain proteins have a deep, hydrophobic pocket they specifically bind the small, lipophilic abscisic acid molecule. Hormone binding triggers a conformational shift described as a ‘gate-latch-lock’ mechanism, in which flexible surface loops enclose the ligand within the pocket (Melcher et al. 2009). This closed state creates a docking interface that binds and inactivates type 2C protein phosphatases. These phosphatases are negative regulators that otherwise suppress stress-signalling pathways (Joshi-Saha et al. 2011).

*Arabidopsis thaliana* possesses 14 distinct cytosolic ABA receptors. This functional redundancy likely serves to fine-tune physiological and tissue-specific responses while expanding the dynamic range of stress signalling (Pri‐Tal et al. 2024).

### Structural and functional insights into bacterial START-domain proteins

Although START domains were first characterized in eukaryotes, comparative genomic analyses show that they are also widely distributed across bacteria, including both nonpathogenic and pathogenic species (Schrick et al. 2004, 2014). Most bacterial START proteins occur as single, soluble domains with low sequence identity to their mammalian counterparts, yet they preserve the characteristic helix-grip fold, pointing to deep evolutionary conservation (Thorsell et al. 2011). Functional studies remain limited, but current evidence suggests roles in lipid binding, small-molecule sensing, and catalysis (Iyer et al. 2001, Li et al. 2022). Together, these findings indicate that, although the core fold is conserved, bacterial START domains have adapted to distinct metabolic requirements. This evolutionary context forms the basis for examining the structural features that differentiate bacterial START domains from their mammalian counterparts.

**Figure 5:**
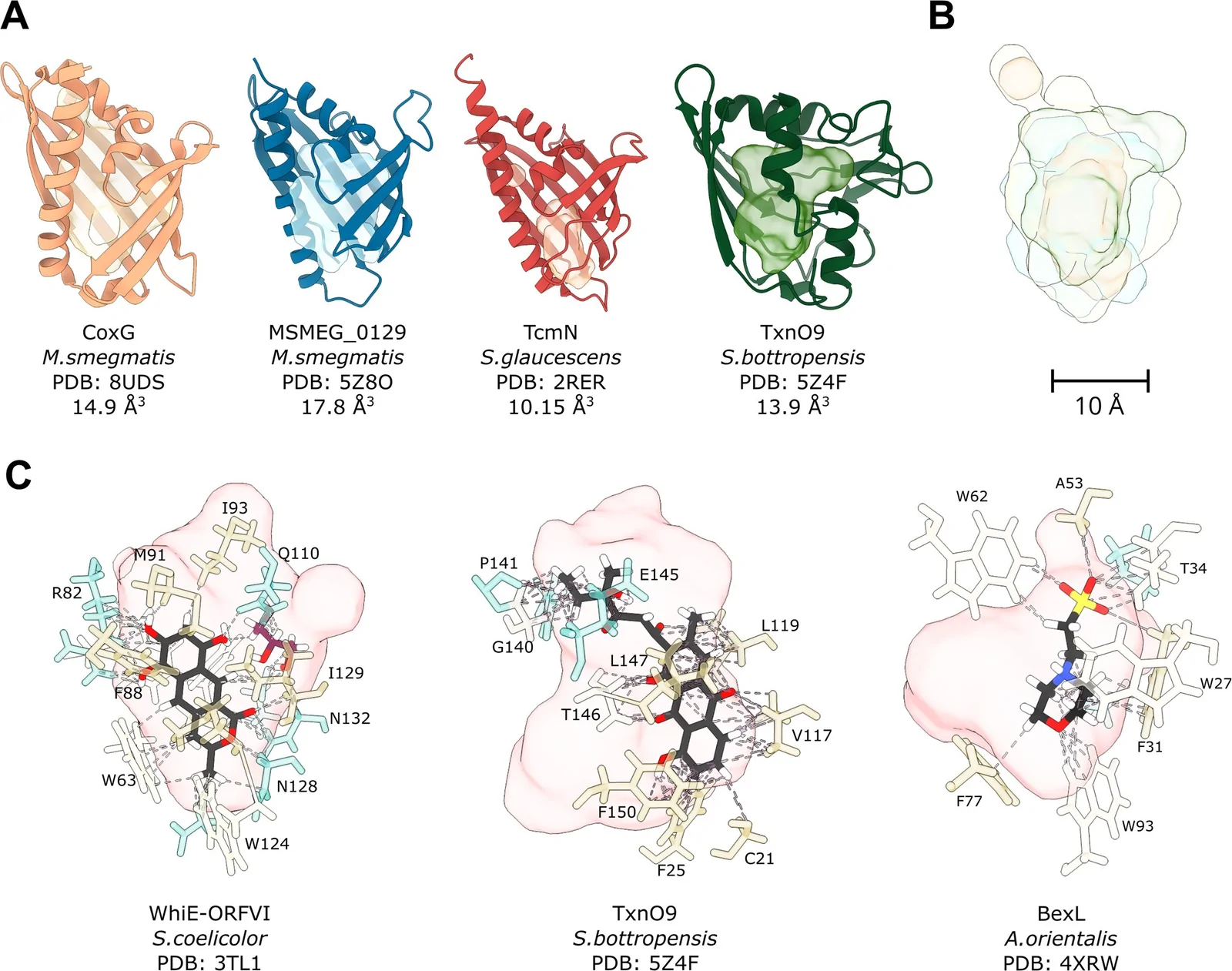
Structural diversity of ligand-binding cavities in bacterial START-domain proteins. (A) Representative bacterial START-domain proteins illustrating the diversity of ligand-binding cavity size and shape. Cavities are shown as coloured surfaces, with the corresponding cavity volume (Å³) indicated for each protein. (B) Structural superposition of the ligand-binding cavities shown in (A), highlighting the conserved protein fold despite substantial variation in cavity geometry. Scale bar, 10 Å. (C) Ligand-binding cavities of bacterial START-domain proteins crystallized in complex with their ligands. Cavities are shown as pink surfaces, while amino acid residues contacting the ligand are coloured according to their physicochemical properties: hydrophobic residues in beige and hydrophilic residues in blue. Cavities were calculated and visualized using parKVFinder v1.2.0 (Guerra et al. 2020).

Despite strong conservation of the α/β helix-grip framework, bacterial START domains show several structural alterations (Fig. 1). Importantly, bacterial START domains are generally shorter (∼140 amino acids instead of ∼200), contain only seven β-strands, and typically lack the N-terminal α-helix (α1) while keeping the central hydrophobic cavity intact (Nakabayashi et al. 2005, Lee et al. 2010). The missing β-strands correspond to β1 and β2, which also means that the two conserved small helices α2 and α3, are now situated between the first and second β-strands (Lee et al. 2010). Importantly, this truncation does not dramatically reshape the internal cavity, some bacterial START domain proteins have cavities with the same size as their mammalian counterparts (Fig. 5). However, some bacterial ligand-binding cavities are considerably smaller, mainly due to bulky residues that obstruct that fill the cavity. Whereas mammalian sterol-binding START domains possess long, continuous tunnels formed by a nine-stranded β-sheet, with cavity volumes of ∼ 873–2297 ${{{\mathring{\rm A}}}^3}$ (Hurley and Tsujishita 2000, Mesmin et al. 2013a, Létourneau et al. 2014b), the START protein from *Thermus thermophilus* (TTHA0849) has an internal pocket of only ∼18 ${{{\mathring{\rm A}}}^3}$ (Nakabayashi et al. 2005). This difference probably reflects the shift from sterol transport towards specialized catalysis or small-molecule binding for some START proteins in bacteria.

The structural differences extend to the α-helical elements that flank the β-sheet core. Mammalian START proteins possess a longer, dynamic C-terminal helix that could function as a movable lid, undergoing conformational changes to allow ligand entry (Hurley and Tsujishita 2000, Schrick et al. 2004, Horenkamp et al. 2018). Bacterial START domains usually contain a shorter C-terminal helix that does not seem to function as a gating lid (Nakabayashi et al. 2005, Stark et al. 2010a). The reduction of both N- terminal helical element in bacterial START proteins also means that the amphipathic, membrane-interacting surface that characterizes mammalian lipid-transfer START domains (Stark et al. 2010b, Mesmin et al. 2013a) is missing. Mammalian START proteins rely on their extended C-terminal helix and Ω-loops to form hydrophobic docking surfaces for nonvesicular lipid transport, enabling interaction with donor and acceptor membranes. In contrast, bacterial START domains are mostly soluble cytosolic proteins, unless they are fused to multiple transmembrane helices, such as the CAS-type START protein of *Pseudomonas aeruginosa* and CoxG of *Mycobacterium smegmatis* (Iyer et al. 2001, Zhou et al. 2020, Kropp et al. 2025a). The large flexible Ω1 loop located between β5 and β6 is conserved in bacterial START domain proteins and possibly this loop forms the main entrance to the cavity.

Despite their wide genomic distribution, a limited number of bacterial START proteins have been studied experimentally, meaning that the vast majority of bacterial START domains remain functionally unexplored. Based on structural analyses and the known activities of related superfamily members, bacterial START domain proteins are generally thought to fall into three functional categories: (i) enzymes, (ii) binding/transfer of hydrophobic ligands, and (iii) regulatory or stress-associated functions. These categories reflect different ways in which the conserved helix-grip fold can be adapted to support survival in varied bacterial environments, although many proposed functions remain to be experimentally confirmed.

Within this first category, bacterial START proteins can function as catalysts that act on diverse small-molecule intermediates. Importantly, the initial description of the START superfamily proposed that this fold could support at least two types of enzymatic activity: cyclase/aromatase and RNase activity, though the RNase activity remains rare and poorly characterized (Iyer et al. 2001)^.^ In a number of bacteria, START-fold proteins have diversified into cyclase/aromatase-type START domains (CAS), which direct the conversion of polyketide intermediates into stable aromatic scaffolds (Caldara-Festin et al. 2015). This catalytic repurposing might provide evolutionary context for why certain bacterial lineages, such as the Actinomycetota, show large expansions of START homologs linked to natural-product biosynthesis This class is described in more detail in the next section.

A second functional category lies in the binding and intracellular transport of hydrophobic ligands. Many bacterial START domain proteins resemble the mitochondrial COQ10 proteins of eukaryotic cells. This could mean that ubiquinone binding and transport in bacteria are dependent on this protein family. Indeed, there is some evidence that in *Escherichia coli* and *Caulobacter crescentus* the Coq10 homologue (PasT cq CC1736) is thought to bind ubiquinone within a narrow hydrophobic tunnel and deliver it to its appropriate sites in the electron transport chain, functioning as dedicated intracellular ‘lipid couriers’ for this redox-active metabolite (Shen et al. 2005, Fino et al. 2020). The strong functional conservation, which is illustrated by the rescue of *E. coli* ΔpasT respiration defects by human COQ10A, underscores that these START domains primarily act as ligand-trafficking modules. This functional characterization of PasT corrects earlier hypotheses that the *E. coli* PasTI locus encoded a toxin–antitoxin (TA) system (Fino et al. 2020). The prevalence of Coq10-like START proteins across diverse bacterial genomes suggests that this type of small lipophilic cofactor-specific intracellular transport could be widespread and form an ancient functional specialization of the bacterial START fold proteins.

Then there are START proteins such as TTHA0849 from the thermophile *T. thermophilus* that possess unusually small ligand-binding cavities (Nakabayashi et al. 2005). In high-temperature environments where hydrophobic metabolites risk aggregation or thermal degradation, compact START domains may function as stabilizing chaperones, sequestering small hydrophobic fragments or metabolic intermediates during biosynthesis or repair. On the other hand, bacterial START domain proteins with tiny cavities are also found in nonthermophiles such as *M. tuberculosis* (see below).

In mycobacteria, there is a protein that combines a START domain with a tiny cavity with a classical DNA-binding motif (Rv0576). Overexpression of this protein (Rv0576) resulted in the differential expression of 30 different genes (Rustad et al. 2014). Possibly, the START domain functions as a sensing domain for specific hydrophobic compounds. Recently a completely different regulatory bacterial START domain protein was identified in *Xanthomonas citri*. This bacterium is the causative agent of citrus canker, a highly contagious characterized by brownish lesions on leaves, stems, and fruit resulting in defoliation and premature fruit drop. A central virulence factor is the type III secretion system (T3SS) of *X. citri*. Interestingly, mutants lacking the START domain protein also failed to produce canker symptoms on host plants, indicating a complete loss of virulence (Zhu et al. 2025). This regulatory protein has a signal sequence, followed by a START domain and a GyrI-like small molecule binding domain. Complementation with only the signal sequence and the START domain restores pathogenicity, demonstrating that the C-terminal GyrI domain is not required for activity. Deletion of the corresponding gene blocks the induction of two crucial T3SS regulatory genes *hrpG* and *hrpX*, and, as a result, the T3SS machinery components and the genes encoding effector proteins. The direct role of the START protein in the regulatory pathway is not fully elucidated, but deletion results in a major shift in a number of metabolites, indicating that it could be involved in sensing environmental cues and, in response, produce a secondary signal (Zhu et al. 2025). A START domain has also recently been reported in Cystatin polymorphic toxins, a distinct family of bacterial toxin systems, suggesting that recruitment of the fold for regulatory or toxin-associated functions may be more common among bacteria than previously known (Burroughs et al. 2025).

Outside of the three major functional categories discussed above, additional roles for bacterial START domains have emerged from studies of specialized natural-product biosynthetic systems, particularly those associated with antibiotic resistance. One of the clearest examples is found in the enediyne-producing Actinomycetes, where the START-fold protein CalC functions as part of an intrinsic resistance strategy against the potent DNA-cleaving antibiotic calicheamicin (Singh et al. 2006). CalC accommodates the aryl tetrasaccharide portion of calicheamicin and positions the reactive enediyne moiety for controlled chemical inactivation. Notably, this illustrates the broader ligand specificity that the START fold can support. The mechanism by which CalC confers resistance follows a self-limiting ‘suicide’ model, in which the protein undergoes oxidative proteolysis initiated by hydrogen abstraction at a defined glycine residue upon binding the antibiotic (Singh et al. 2006). This reaction disables the calicheamicin warhead while simultaneously degrading the CalC protein itself, thereby preventing the metabolite from damaging cellular DNA. Structural analysis also revealed a small DNA-interacting region, suggesting that CalC may act in proximity to the antibiotic’s site of action, although its primary functional contribution lies in direct sequestration and controlled inactivation of the reactive molecule (Singh et al. 2006). It is not clear yet if this is the only and primary function of CalC or a fortuitous additional function.

In summary, bacterial START domains appear to form a versatile functional group, with potential roles that may span specialized enzymatic catalysis, the intracellular handling of hydrophobic metabolites and participation in stress-associated regulatory processes. Among these putative functions, the cyclase/aromatase-type START domains represent the most striking example of functional divergence from mammalian START proteins.

### Bacterial polyketide cyclases defined by START domains

In bacteria, the START domain proteins known as CAS have evolved enzymatic functions integral to secondary metabolite biosynthesis (Iyer et al. 2001, Lee et al. 2012, Zhou et al. 2021). The fundamental biochemical role of CAS domains is associated with type II polyketide synthase (PKS) systems, where they catalyse cyclization and aromatization reactions during the maturation of linear poly-β-ketone intermediates (Caldara-Festin et al. 2015) .Within this context, the START domain promotes intramolecular cyclization, followed by dehydration steps required to establish aromatic ring systems characteristic of many bacterial polyketide natural products. The end products are usually secondary metabolites such as antibiotics, pigments, and toxins (Iyer et al. 2001). The mechanism relies on the α/β helix-grip fold, which generates a deep internal hydrophobic pocket that constrains the flexible polyketide chain into a productive conformation (Ames et al. 2008). Orientation of the substrate within this pocket largely determines the regioselective cyclization pattern, including commonly observed C9–C14 or C7–C12 modes. Specific polar or charged residues lining the pocket anchor the substrate and stabilize reaction intermediates, enabling the sequence of catalytic steps performed by the domain. Three residues, that are all facing the cavity, have been indicated as active site residues, an Arg, present in β-sheet 5, a His/Gln on β-sheet 2 and a variable hydrophilic amino acid on the C-terminal α-helix (Ames et al. 2008, Caldara-Festin et al. 2015). These enzymes are frequently found in *Streptomyces* (Iyer et al. 2001, Ames et al. 2008, Lee et al. 2012) and occur in both mono-domain and di-domain architectures. This structural variation correlates strongly with their functional specialization. Mono-domain ARO/CYCs, such as TcmN and WhiE, contain a single helix-grip START fold whose deep internal pocket governs substrate binding, chain-length discrimination, and regiospecific first-ring cyclization (Ames et al. 2008, Lee et al. 2012). In these enzymes, the START fold alone is sufficient to catalyze the initial ring-forming steps (typically the C9–C14 or C7–C12 aldol condensations) through a conserved residues including Tyr35, Arg69, and Arg82 that anchor and fold the polyketide into the correct conformation (Ames et al. 2008). In contrast, di-domain ARO/CYCs of *Streptomyces* such as StfQ and BexL consist of two START domains that have diverged to perform distinct roles within nonreducing and reducing PKS pathways, respectively (Caldara-Festin et al. 2015). In StfQ, which acts on a fully unreduced poly-β-ketone chain, catalytic activity resides predominantly in the C-terminal domain, where residues such as Arg218 promote C7-C12 aldol cyclization, while the N-terminal domain contributes mainly to structural stabilization (Caldara-Festin et al. 2015). Conversely, in the reducing PKS cyclase BexL, the catalytic center is located in the N-terminal domain, which carries out dehydration of the C9 hydroxyl group and subsequent first-ring aromatization on a prereduced intermediate, whereas the C-terminal domain is largely dispensable for catalysis (Caldara-Festin et al. 2015).

Beyond the classical cyclase/aromatase-type reactions, some bacterial START-domain enzymes have adopted even more specialized catalytic roles within polyketide maturation pathways. A notable example is TxnO9 from *Streptomyces bottropensis*, which participates in the biosynthesis of the antitumor antibiotic trioxacarcin A (Hou et al. 2018). TxnO9 was identified as an unexpected member of the START superfamily, expanding the known catalytic repertoire of this fold into heterocyclization chemistry. Unlike canonical ARO/CYC enzymes that mediate aldol-type cyclizations of linear poly-β-ketones, TxnO9 catalyses two post-PKS tailoring reactions on an unstable biosynthetic intermediate: an intramolecular C-O bond-forming heterocyclization followed by dehydration. These steps generate an anthraquinone-γ-pyrone scaffold that subsequently undergoes spontaneous rearrangement, producing the characteristic four-ring structure of trioxacarcin precursors. The discovery of TxnO9 thus establishes a new subclass of polyketide heterocyclases within the START domain superfamily. Structurally, TxnO9 differs from both mammalian START proteins and well-characterized bacterial polyketide cyclases (Zhang et al. 2015, Hou et al. 2018). NMR analysis shows that the protein retains a hydrophobic internal cavity, but has an expanded set of peripheral loops and eight antiparallel β-strands surrounding the pocket (Hou et al. 2018). Functional studies, including gene deletions and site-directed mutagenesis, identified key residues such as Lys10, Glu145, and Thr146 as essential for turnover (Hou et al. 2018). Thr146 activates the C-13 carbonyl group of the substrate through hydrogen bonding, initiating the heterocyclization reaction. These features highlight the extent to which the START fold can be structurally adapted for distinct catalytic functions.

### START-domain proteins in *M. tuberculosis*

**Figure 6:**
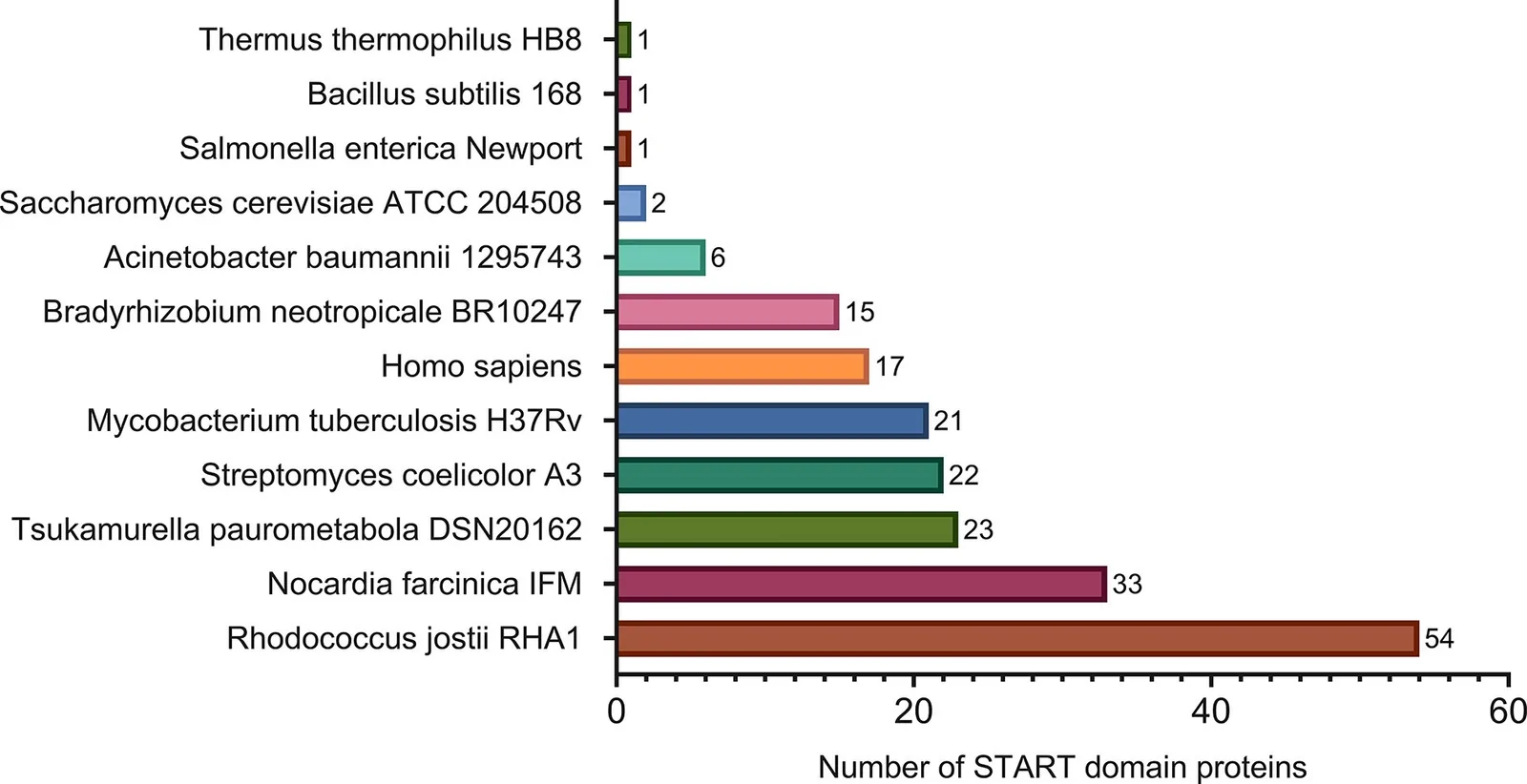
Comparative abundance of START domain proteins across representative bacterial species. Number of proteins containing a START-like domain identified in representative genomes spanning bacteria, fungi, and mammals, based on the InterPro ‘START-like domain superfamily’ entry (IPR023393). Actinobacterial genomes, particularly *R. jostii* RHA1 (*n* = 54), *N. farcinica* IFM (*n* = 33), and *Tsukamurella paurometabola* DSN20162 (*n* = 23) contain expanded START-domain proteins relative to other bacteria and to *Homo sapiens* (*n* = 15), while several non actinobacterial species encode only a single START-domain protein.

Genomic surveys show that most bacterial strains, such as *T. thermophilus* HB8, *Bacillus subtilis* 168, and *Salmonella typhimurium* Newman each encode only a single START-like protein. This is in stark contrast to many Actinomycetota, which show a dramatic expansion of START domain proteins: *Streptomyces coelicolor* A3 has 22, *Nocardia farcinica* IFM has 33, and *Rhodococcus jostii* RHA1 has a staggering number of 54 START domain proteins (Fig. 6). This pattern indicates that actinomycetes, more than any other bacterial group, has diversified the START-like fold to support sophisticated lipid-dependent physiology and/or polyketide-dependent synthesis of secondary compounds. This lineage-specific proliferation might suggest that START domains have become integral components of lipid-responsive signalling networks in Actinomycetota, likely enabling fine-tuned coordination between metabolism, envelope biogenesis, and environmental adaptation. Their diversification may reflect evolutionary pressure to sense and regulate an unusually wide repertoire of hydrophobic metabolites, including mycolic acids, isoprenoids, and other complex lipids essential for virulence and stress tolerance (Nataraj et al. 2015, Carvalho de et al. 2016). The evolutionary expansion of START domains in these species is not merely a quantitative observation but might be a signature of lipid-centric cellular strategies that define actinomycete biology. We have chosen to describe all START proteins of *M. tuberculosis* (Mtb) as a representative of this phylum. Mtb is probably the best studied actinomycete and has the highest impact on society and a number of its START domain proteins have been studied in detail.

We compared all 21 START domain proteins of *M. tuberculosis* (as listed in the UniProt database, see Table 1) and noticed a couple of universal features. Each member of this family retained the characteristic α/β helix-grip fold enclosing a hydrophobic cavity, only one seems to be a bit smaller with only five predicted β-strands (Rv2186c). The Mtb START proteins display simple domain architectures, i.e. they consist of only a START domain, except for Rv0369 (CoxG homologue) and Rv0576. The first one has an additional C-terminal trans-membrane domain (TMD) that connects it to the cytoplasmic membrane and is further discussed below. Rv0576 has two additional domains, one putative DNA-binding domain, indicating a regulatory function, and a four-helix bundle with unknown function. This also means that all other Mtb START domain proteins lack predicted signal sequences, suggesting that they are all cytosolic. This indicates that, if these proteins are involved in lipid transport, it would have to be in the process of lipid body formation and not in shuttling between the inner and mycobacterial outer membrane. However, analysis of Mtb proteins associated with lipid droplets failed to identify thus far any START domain protein (Low et al. 2010), which further lowers the chance that any START domain protein is a lipid transport protein similar to the mammalian homologues. Perhaps they are more involved in sensing of specific hydrophobic ligands, such as the abscisic acid receptors in plants. We also determine the internal cavity based on Alphafold structure prediction combined with parKVFinder software. This analysis shows that, despite the overall similar fold, the differences in cavity size is very high. Many mycobacterial START domain proteins (9 out of 21) have cavities in the same range as mammalian START domain proteins, indicating that they can bind lipids or lipophilic molecules such as polyketides or ubiquinone/menaquinone. Then, there are six members with a larger cavity, which can be even more than double the size (Rv0369c/CoxG). This effect is mainly caused by the high number of Ala, Val, and Gly residues in these proteins that are facing the inside of the β-sheet, whereas members with small cavities have more bulky hydrophobic amino acids (Leu and Phe). Finally, there are also six START proteins with a very small cavity. Rv2186c belongs to this group, which is perhaps not surprising as it has only five predicted β-sheets. However, the other members with small cavities have a normal START domain fold and are limited by the presence of bulky amino acids. The size of their cavities will probably not allow lipids or even menaquinone/polyketides to bind and possibly their function is more regulatory.

**Table 1:** Comparative overview of all START domain proteins in *M. tuberculosis*.

Protein	Operon	Essential	Function	Extra domain	β-strands	α-helices	Cavity volume (Å^3^)
Rv0088	No	No	Unknown	No	7	3	600
Rv0164 (TB18.5)	No	Yes	Unknown	No	7	3	510
Rv0369c	rv0368c	No	CoxG (menaquinone)	Yes, C-terminal TMD	7	3	1364
Rv0481c	No	No	Unknown	No	7	3	671
Rv0576	TB27.3 (CFP32)	No	Regulatory	Yes, 2 domains			111
Rv0854	Far	No	Unknown	No	7	3	798
Rv0856	No	No	Unknown	No	6	3	945
Rv0857	No	No	Unknown	No	7	3	738
Rv0910	rv0909	No	Acyl-AcpM binding	No	7	3	337
Rv1060	rv1061/rv1062	No	Unknown	No	7	3	459
Rv1443c	No	No	Unknown	No	7	3	470
Rv1546	No	No	RNase	No	7	3	538
Rv1883c	rv1882c	No	Unknown	No	7	4	543
Rv1919c	No	No	Unknown	No	7	3	229
Rv2035	rv2034/rv2035/rv2036	No	Unknown	No	7	3	139
Rv2185c/TB16.3	No	No	Unknown	No	7	3	848
Rv2186c	No	No	Unknown	No	5	4	25
Rv2574	rv2575	No	Unknown	No	7	3	267
Rv2778c	No	No	Unknown	No	7	4	563
Rv3076	rv3077/rv3078	No	Unknown	No	7	3	68

The table summarizes the annotated START and START-like domain proteins encoded by *M. tuberculosis* H37Rv, including their Rv numbers, putative functions, operonic structure, essentiality, Alphafold predicted structure (number β-sheets and α-helices), and cavity based on Alphafold structure prediction combined with parKVFinder software. Essentiality is based on high-density transposon mutagenesis. Operonic structure is predicted based on genome analysis and transcription profiles.

Different transposon mutagenesis studies in Mtb and closely related bacteria have shown that only a single one of these proteins is essential, Rv0164 (also known as Tb18.5). This protein was recently shown to be a promising drug target (see below). A small majority of the genes (12) do not seem to be organized in an operon (MTB Genome Browser. https://mtb.wadsworth.org/), which complicates function prediction. Those that appear to be part of an operon are usually in a small operon with one or two other genes (Table 1). The function of the coregulated genes are widely variable, but in most cases enzymes, ranging from putative metallopeptidase to fatty acid CoA racemase and glutamine amidotransferase. These features, combined with the deep evolutionary origin of the START superfamily, indicate that mycobacterial START proteins represent ancient, structurally conserved elements that have been adapted primarily toward metabolic functions. RNAseq and ribosome mapping experiments indicate that most of the START domain encoding genes (possibly excluding two) are transcribed under normal laboratory conditions (Shell et al. 2015). Phylogenetic analysis of the Mtb START proteins with selected Actinomycetota species indicates that most of the *M. tuberculosis* START domains are not due to a recent expansion but have been around in common ancestors. The only exceptions are the START proteins Rv0854, Rv0856, and Rv0857, whose genes occur in close genomic proximity; especially Rv0856 and Rv0857 seem to be recently duplicated.

### Therapeutic potential of the Rv0164 START domain protein

Rv0164 is not only the only essential START domain protein in *M. tuberculosis* and also the closely related species *Mycobacterium marinum* according to genome-wide transposon screens and also genetical analysis (Habjan and Speer, unpublished results). Two different classes of compounds both block Rv0164, probably by binding inside the cavity, and efficiently block the growth of *M. tuberculosis* (Tsui et al. 2022, Habjan et al. 2024). Notably, one study showed that these compounds show synergy with larger antibiotics, such as rifampicin and vancomycin and additional assays showed that this effect is due to increased cell envelope permeability (Habjan et al. 2024). This probably means that the composition of the mycobacterial outer membrane has been compromised by blocking this protein, indicating that Rv0164 could be involved in the production of mycobacterial lipids or the regulation of enzymes involved in this process. Alternatively, the incorporation of external lipids could be compromised, as the antibacterial effects were stronger in the presence of fatty acids in the medium or upon infection of host cells (Habjan et al. 2024). These results do show that START-domain regulation of envelope lipids represents a promising antimicrobial target (Tsui et al. 2022, Habjan et al. 2024). While these findings strongly support a role for Rv0164 in envelope maintenance and antibiotic susceptibility, the precise lipid species or downstream enzymatic pathways affected by Rv0164 inhibition remain unresolved. The essentiality and conservation of Rv0164 across mycobacteria both support its identification as a druggable metabolic node, further strengthened by findings showing its inhibition increases outer membrane permeability in related species (Habjan et al. 2024).

The orthologue of Rv0164 in *M. smegmatis* (MSMEG_0129) displays a rapid turnover and experimentation seems to indicate that this protein interacts with central regulatory factors, including the ClpP2 protease and the transcription factor CarD (Zhou et al. 2020, Habjan et al. 2024). This behaviour supports a model in which ligand occupancy of the START fold modulates protein conformation and, consequently, transcriptional accessibility. In another study the transcriptome of *M. smegmatis* with either reduced or overexpressed levels of MSMEG_0129 was determined. These authors showed that changes in the amount of this START protein affect the transcription of ~3% of protein-coding genes, including activation of *desA1* and modulation of SigF regulon via the anti-SigF antagonist MSMEG_0586 (Zhou et al. 2021). The gene *desA1* encodes an essential acyl-ACP desaturase required for introducing double bonds during mycolic acid biosynthesis, a process fundamental to maintaining cell envelope integrity and the physicochemical properties of mycobacterial lipids (Singh et al. 2016). SigF is an alternative sigma factor associated with transitions to slow growth and adaptation to stress conditions, such as oxidative stress, nutrient limitation, and the stationary phase (DeMaio et al. 1996, Hümpel et al. 2010). However, the molecular mechanism by which the START domain protein would influence gene regulation has not been identified.

Structural analyses further clarify the non-catalytic regulatory role of Rv0164. Rv0164 is frequently annotated as a COQ10-like START protein or as a putative cyclase. However, comparison with polyketide cyclase/aromatase members reveals that Rv0164 lacks the catalytic residues (including the conserved Arg and pair of tryptophan residues) that define CYC/ARO enzymes (Zheng et al. 2018), indicating that this protein is noncatalytic. The resolved structure of MSMEG_0129 contains a deep hydrophobic cavity that has good plasticity, indicating that a larger lipid or lipophilic molecule could be bound. In the case of MSMEG_0129 an unidentified molecule resembling four planar rings was identified, but this was deemed an artefact from growing the bacteria for prolonged time on a plastic tray.

### Redefining the function of Rv0910

Like the PasT START domain protein of *E. coli*, also Rv0910 was labelled as a toxin and part of a TA pair. Experimental evidence showed that overexpression of the *M. smegmatis* orthologue of *rv0910* was lethal to the cells, which could be rescued by coexpression of neighbouring gene *rv0909*. The mode of action was less clear, as Rv0910 does align with START domains with enzymatic activity, but it lacks detectable RNase activity (Ramage et al. 2009).

Rv0909–Rv0910 was originally characterized as a TA pair, with expression of Rv0910 inhibiting growth and this effect reversed by coexpression of Rv0909; the *M. smegmatis* orthologues MSMEG_5635–MSMEG_5634 behaved the same way (Ramage et al. 2009). More recently, this TA activity has been questioned. In a recent publication, the toxic effect was not observed and the *M. smegmatis* orthologues MSMEG_5634 and MSMEG_5635 did not interact with each other (Li et al. 2022). Furthermore, the authors showed that the Rv0910 orthologue of *M. smegmatis* (MSMEG_5634) plays a role in mycolic acid biosynthesis. MSMEG_5634 binds long-chain acyl-AcpM intermediates of the FAS-II system (Li et al. 2022). Its activity depends on the presence of a fatty acyl chain and modulates fatty acid elongation by sequestering acyl-AcpM from the FAS-II complex. By doing so it influences susceptibility to isoniazid and triclosan antibiotics (Li et al. 2022). This illustrates how cytosolic START domains contribute to lipid synthesis and antibiotic sensitivity without participating in long-range lipid transport. These conflicting observations suggest that Rv0910 may not function as a classical toxin but instead represents a START-derived regulatory protein, whose apparent toxicity may depend on expression context. Possibly also other START domain proteins are required to keep lipid intermediates accessible for cytosolic enzymes.

### Rv0369c, an old dog with a new trick

Recently, an exciting new study was published on the *M smegmatis* START domain protein CoxG, a close homologue of Rv0369c. This is the START protein with the largest predicted cavity. This study was able to explain how some bacteria oxidize CO at extremely low concentrations and connect this process mechanistically to their energy metabolism (Kropp et al. 2025a). *Mycobacterium smegmatis* uses an enzyme complex called molybdenum–copper CO dehydrogenase (Mo–CODH) to oxidize atmospheric CO to carbon dioxide (CO₂) (Cordero et al. 2019). This complex consists of three proteins, CoxL, CoxM, and CoxS. The oxidation of atmospheric CO is coupled to electron transfer to menaquinone (which is the preferred substrate of the electron transport chain in mycobacteria over ubiquinone). But the Mo–CODH complex can not do this on its own. Here is where CoxG steps in. With its START domain that is reminiscent of the COQ10 protein it can efficiently bind menaquinone (Cordero et al. 2019, Kropp et al. 2025b). Alphafold structure predictions show that CoxG can bind the CoxLMS enzyme complex. Five positively charged residues on the interaction site that were predicted to pair with negatively charged residues of the CoxLMS complex were shown to be essential for enzyme activity, reinforcing this prediction. At the same time CoxG is anchored to the membrane by its C-terminal TMD. Mechanistically, CoxG is thought to shuttle menaquinone from the membrane to the CoxLMS enzyme complex and then, once the reaction has taken place, bring back the reduced form, menaquinol, to the membrane, where it can function in the electron transport chain to provide electrons to the terminal oxidases (Kropp et al. 2025b). This interaction enables efficient electron transfer from CO oxidation into the bacterial respiratory chain. Mtb has a close homologue for CoxG (although the start site is misannotated in the type strain H37Rv) and it also has CoxLMS homologues. As such, it seems likely that Mtb can use CO for energy metabolism. Future studies must indicate whether this elegant system with a START domain protein providing menaquinone/ubiquinone to a soluble enzyme complex is also used for other enzyme systems, but in Mtb there is no other START domain protein with a TMD.

### Rv1546, the odd one out or the start of a new dogma

A subset of Mtb START proteins have been classified in cyclase/aromatase-type proteins, despite many lacking conserved catalytic residues. However, loss of conserved catalytic motifs does not necessarily imply functional loss; instead, these proteins may act as structural scaffolds, bind pathway intermediates, or modulate the spatial or temporal organization of lipid biosynthetic processes. Rv1546 exemplifies a completely distinct evolutionary trajectory and could open a new paradigm for START domain proteins. This protein has a predicted classical α/β helix-grip fold with a cavity similar to the cyclase and STARD proteins. However, when they purified the protein, they discovered that it forms both monomers and dimers (Kim et al. 2023). The structure of the dimer was resolved and shown to retain the helix-grip fold, but it had swapped the entire C-terminal α-helix to the other protein molecule. This new structure had hardly any cavity left. This structural rearrangement is directly linked to its ribonuclease activity; a narrow aperture is formed between the two subunits that could bind RNA. Several active site residues have been indicated (Arg63, Lys84, Lys88, and Arg113) that are facing outwards form a monomer point of view, which is completely opposite from the active site residues of the cyclases described previously (Kim et al. 2023). Rv1546 may therefore represent a catalytic outlier within the Mtb START portfolio or form a new paradigm on how START proteins can be adapted to different needs. This demonstrates that START-derived architectures can support enzymatic functions when the canonical internal cavity is absent. Because both monomers and dimers of Rv1546 seem to be stable, one interesting possibility is that the monomer is stabilized by the presence of its substrate, as it seems to have a normal binding pocket, but when this substrate is lacking it starts to form dimers and reveal its RNase activity. Alphafold predictions indicate that possibly also some of the other mycobacterial START domain proteins could form such dimers.

## Conclusion and discussion

The START domain represents a structurally conserved α/β helix-grip fold that has been retained in both eukaryotic and prokaryotic organisms (Iyer et al. 2001, Schrick et al. 2004). In mammals, this domain has been extensively characterized as a nonvesicular lipid transfer module enabling the movement of bulky lipids such as sterols and ceramides within MCSs (Thorsell et al. 2011, Alpy and Tomasetto 2014, Clark Barbara and Stocco 2014, Clark Barbara 2020). This early observation might have skewed our view and resulted in a literature bias heavily focused on mammalian systems. In contrast, the roles of START domains in bacteria have received limited attention. Interestingly, some of the functionally characterized bacterial proteins do contain START domains yet are often not labelled as such but are automatically annotated with diverse terms as polyketide cyclases, aromatases, coenzyme Q-binding protein, or toxin. This inconsistent annotation fragments the field and hinders a unified understanding of START protein biology.

Despite limited characterization, what is known supports a compelling biological narrative. Structural comparison reveals that bacterial START proteins maintain the conserved helix-grip topology but feature a truncated N-terminus and lack the extended C-terminal gating helix that in mammals allows the transfer of bulky lipids (Nakabayashi et al. 2005, Shen et al. 2005, Mercier et al. 2009, Stark et al. 2010a). This architectural shift reduces flexibility. Instead, bacteria appear to have repurposed the fold for diverse roles including enzymatic activity, hydrophobic ligand binding, and regulatory functions (Singh et al. 2006, Fino et al. 2020, Zhou et al. 2020, 2021). The persistence of this fold across billions of years of divergence implies that its ability to bind small hydrophobic molecules remains critical for cellular fitness.

Additional bacterial START variants extend the functional range of the fold even further. CAS-type proteins contribute to chemically precise cyclization and aromatization reactions in polyketide biosynthesis, while the domain swapping Rv1546 protein with its surface-based RNase activity demonstrates a completely new evolutionary road for these proteins (Kim et al. 2023). The cumulative picture is that structural constraints of the START fold show high adaptability. This versatility is likely why the fold has survived intact through evolution. Yet, despite these compelling biological connections, the field remains at an early stage in defining bacterial START functions. The most striking gap is ligand identification. For the majority of *M. tuberculosis* START proteins, the native hydrophobic metabolite remains unknown. Without ligand assignments, it is difficult to establish how these proteins sense and respond to environmental shifts. Furthermore, the reliance on static crystal structures of isolated domains obscures the dynamic conformational changes essential for lipid handling. Without structural data in a membrane-bound context, we lack insight into the transitional states that facilitate substrate loading and unloading. Given the significant energetic barrier of extracting a hydrophobic ligand from a deep protein pocket, these transitional shifts, potentially mediated by auxiliary proteins, are likely the primary determinants of lipid transport efficiency.

Current functional studies also rely heavily on heterologous systems such as *M. smegmatis* or *E. coli*, creating uncertainty about whether phenotypes reflect native conditions. Mycobacteria experience multiple stresses during infection, hypoxia, acidity, and nutrient starvation, that cannot easily be replicated in *in vitro* environments (Vilchèze et al. 2022). As a result, it is plausible that critical functions of START proteins remain cryptic under laboratory growth conditions. Functional redundancy may further complicate interpretation: with 21 paralogs, genetic loss of a single START protein may be buffered, masking essentiality in standard screens. The emerging functional diversity of bacterial START-domain proteins also raises the question of their suitability as pharmacological targets. Importantly, proof-of-concept for pharmacological targeting of START domains already exists in mammalian systems. Natural ligand-nonmimetic inhibitors have been successfully developed against the ceramide transfer protein CERT (STARD11), where compounds identified by virtual screening bind the START domain without mimicking ceramide, instead engaging the lipid-binding cavity through stabilizing hydrogen-bond networks (Nakao et al. 2019). Optimization of these scaffolds yielded potent inhibitors that effectively block CERT function in human cells. These studies demonstrate that START domains are druggable using nonlipid-mimetic strategies, strengthening the rationale that similar approaches could be extended to bacterial START proteins. Bacterial START domain proteins also start to show promise for therapeutic intervention. The best example is the blocking of Rv0164 by small-molecule ligands that can sensitize *M. tuberculosis* to large antibiotics but is also a promising antibacterial compound on its own (Habjan et al. 2024).

## Supplementary Material

fuag042_Supplemental_Files
