# Supplementary material for: Getting a grip on StAR-related lipid-transfer (START) domain proteins in bacteria
Source: FEMS Microbiol Rev. 2026 Aug 22;50:fuag042. doi: 10.1093/femsre/fuag042 (PMC13525633; doi:10.1093/femsre/fuag042)
Supplement: Supplementary file 1 [file fuag042_supplemental_files.zip › Table_Mammalian_STARTs_PDBs.docx]

| **Protein** | **Structure type** | **Ligand** | **PDB ID** |
| --- | --- | --- | --- |
| STARD1 | Apo (crystal) | - | 3P0L |
| STARD2 | Ligand bound (crystal) | Phosphatidylcholine (PC 16:0/20:4), Dilinoleoylphosphatidylcholine, Dilinoleoylphosphatidylcholine (Se-Met), Palmitoyl-linoleoyl phosphatidylcholine (Se-Met) | 7U9D,1LN1, 1LN2, 1LN3 |
| STARD3 | Apo (crystal) | - | 5I9J |
| STARD4 | Apo (crystal) | - | 6L1D |
| STARD5 | Apo (crystal) | - | 2R55 |
| STARD6 | Apo (solution) | - | 2MOU |
| STARD10 | Apo (crystal) | - | 6SER |
| STARD11 | Apo (crystal) | - | 2E3M |
| STARD11 | Ligand bound (crystal) | C6-ceramide (P2₁2₁2₁), C16-ceramide (P1), C16-ceramide (P2₁2₁2₁), C18-ceramide (P1), C18-ceramide (P2₁2₁2₁) | 2E3N, 2E3P, 2E3O,2E3R, 2E3Q |
| STARD13 | Apo (crystal) | - | 2PSO |
| STARD14 | Apo (crystal) | - | 3FO5 |
| STARD14 | Ligand bound (crystal) | Myristic acid | 6VVQ |
| STARD15 | Ligand bound (crystal) | Coenzyme A | 3B7K, 4MOC |
